# Systemic immune programming in the life course of childhood asthma: a new perspective on monocyte pre-activation and trained immunity

**DOI:** 10.3389/fimmu.2026.1895388

**Published:** 2026-09-15

**Authors:** Ran Ma, Kaiwen Zheng, Yingqian Zhang

**Affiliations:** 1 Graduate School, Hebei Medical University, Shijiazhuang, Hebei, China; 2 Department of Respiratory Medicine, Hebei Children’s Hospital, Shijiazhuang, Hebei, China; 3 Hebei Provincial Clinical Research Center for Child Health and Disease, Shijiazhuang, Hebei, China; 4 Hebei Key Laboratory of Pediatric Allergy and Immunology, Shijiazhuang, Hebei, China

**Keywords:** airway remodeling, childhood asthma, lung-bone marrow axis, LYN-SHIP-1 signaling, metabolic-epigenetic reprogramming, trained immunity

## Abstract

**Background:**

Childhood asthma has traditionally been characterized as a localized chronic airway inflammatory disease, with structural remodeling viewed merely as a downstream consequence of persistent inflammation. However, clinical evidence revealing significant structural changes in the airways of children as young as 2.5 years old challenges this paradigm, suggesting that the developmental origins of asthma are established much earlier through systemic mechanisms. While local adaptive immune cascades undoubtedly amplify mucosal pathology, emerging paradigms emphasize that systemic immune reprogramming plays a foundational role in dictating early structural divergence.

**Objective:**

To address these anomalies, this review proposes the “Systemic Priming and Homeostasis in Pediatric Asthma” (SPHPA) model to elucidate how early-life environmental exposures translate into lifelong pathological imprints.

**Results:**

We highlight the pivotal role of the lung-bone marrow axis, where early-life triggers—such as respiratory syncytial virus or human rhinovirus infections and early-life allergens—induce trained immunity in hematopoietic stem cells (HSCs). This systemic programming pre-activates the monocyte-macrophage lineage prior to their recruitment to the lungs. Central to this process is the LYN-SHIP-1-Trib1 signaling circuit, where LYN acts as a molecular gatekeeper. We discuss the non-canonical “functional flip” of SHIP-1 from an anti-inflammatory inhibitor to a pro-inflammatory scaffold under stressful pulmonary microenvironments, fueled by metabolic reprogramming events such as proline biosynthesis and histone lactylation (H3K18la). Upon homing to the lungs, these primed monocytes undergo pathogenic alternative activation (M2-like macrophage polarization) via the SHIP-1-STAT5-Trib1 circuit, directly driving airway smooth muscle (ASM) hypertrophy, goblet cell hyperplasia, and reticular basement membrane (RBM) thickening even before the establishment of chronic mucosal inflammation.

**Conclusion:**

Understanding systemic monocyte pre-activation provides a novel window for early intervention. Future strategies should shift from localized anti-inflammatory treatments toward resetting systemic innate immune memory using conformation-specific ligands and epigenetic modulators, potentially reversing the trajectory of severe pediatric asthma.

## 1 Introduction

Asthma remains a serious global health problem affecting approximately 300 million people around the world (1). It is a growing burden for patients, communities, and healthcare systems, and it heavily interferes with education and family life, especially when children are affected. Approximately 10% of pediatric patients suffer from severe phenotypes that remain poorly controlled despite high-dose inhaled corticosteroids (ICS) and advanced biologics (2, 3). To address this escalating clinical and public health challenge, establishing a comprehensive understanding of early-life systemic immune programming—such as the SPHPA model—is of critical importance for severe pediatric asthma.

For over three decades, the prevailing paradigm of pediatric asthma research has been dominated by the “Airway-Centric Model,” confining pathogenesis to localized mucosal response (4). Within this model, allergen exposure stimulates T helper 2 (Th2) and group 2 innate lymphoid cell (ILC2) activation, driving eosinophilic inflammation via the secretion of interleukins IL-4, IL-5, and IL-13 (4). Consequently, airway remodeling—including subepithelial fibrosis, goblet cell hyperplasia, and airway smooth muscle (ASM) hypertrophy—is conceptualized as a chronic, downstream consequence of prolonged inflammatory burden. However, mounting clinical and pathological evidence challenges this classical framework. A landmark systematic review demonstrated that airway remodeling—specifically reticular basement membrane (RBM) thickening and increased ASM area—is already fully established in wheezing preschool children as young as 2.5 years of age (5). While this early structural divergence occurring prior to overt persistent mucosal inflammation is an increasingly recognized clinical fact supported by pediatric biopsy cohorts, the conceptual framework explaining why and how this occurs via systemic mechanisms represents a novel theoretical perspective (5). Furthermore, extensive birth cohort studies indicate that a significant proportion of asthmatic children exhibit persistent deficits in lung function trajectories before 3 years of age (6), a developmental window during which the typical Th2-mediated adaptive immune response has not yet fully matured (7). These paradoxes strongly suggest that the developmental origins of childhood asthma are not restricted to localized airway insults, but are governed by deeper, systemic developmental programming aberrations (8).

To reconcile these clinical anomalies, we propose the “Systemic Priming and Homeostasis in Pediatric Asthma” (SPHPA) model. Although alternative explanations attribute early lung function deficits solely to genetic predisposition or environmental triggers, our framework posits that early-life multi-hit exposures operate via a dynamic “lung-bone marrow axis”. This axis induces “trained immunity” within hematopoietic stem and progenitor cells (HSPCs), fundamentally altering monocyte-macrophage trajectories (9, 10). While acknowledging that this model represents an evolving hypothesis rather than a singular universal cause, we identify the LYN-SHIP-1-Trib1 signaling circuit as the key molecular engine of this priming process (11, 12), wherein circulating monocytes are systematically pre-activated (“primed”) prior to their migration into pulmonary tissues (13). Dysregulation of this gatekeeper axis—characterized by a non-canonical, pro-inflammatory “functional flip” of the lipid phosphatase SHIP-1—dictates the pathological fate commitment of monocytes (11), offering a comprehensive mechanistic perspective of the bone marrow-circulation-lung axis to guide novel early-life therapeutic interventions (**Figure 1**).

**Figure 1:**
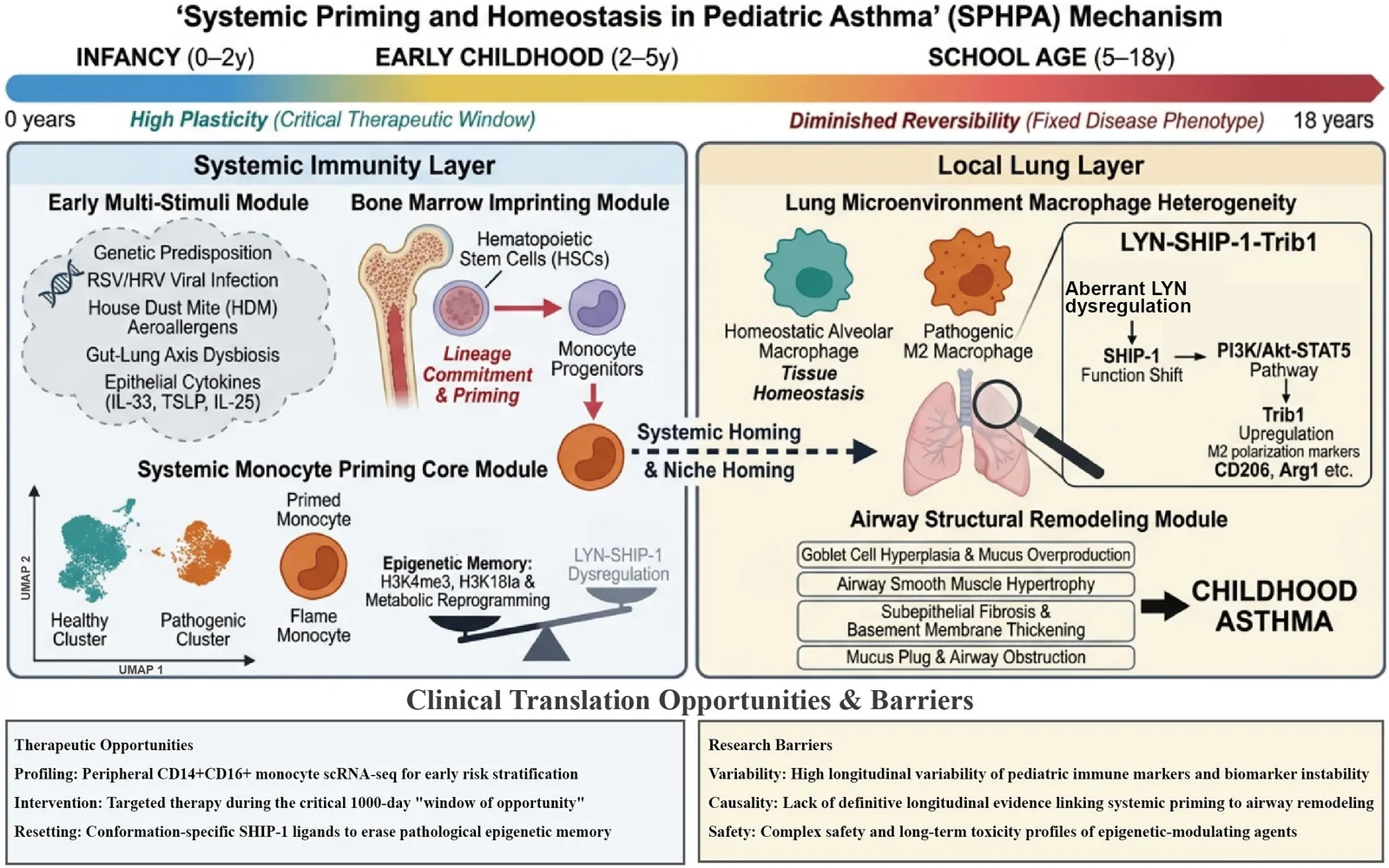
Conceptual and biochemical framework of the ‘Systemic Priming and Homeostasis in Pediatric Asthma’ (SPHPA) model integrating temporal plasticity with systemic and local pathogenesis.

The upper horizontal gradient depicts the temporal evolution of immune and structural plasticity across the pediatric life course, progressing from a ‘Critical Therapeutic Window’ in infancy (0-2 years) toward a ‘Fixed Disease Phenotype’ by school age (5-18 years) due to age-dependent plasticity decay. The Systemic Layer (left panel) delineates extra-pulmonary innate immune programming, whereby genetic predisposition and repeated mucosal insults drive bone marrow HSC imprinting via sustained epithelial alarmin secretion. Acquisition of distinctive metabo-epigenetic memory overrides dysregulated LYN-SHIP-1 homeostatic braking, tilting pre-activated classical/intermediate monocytes toward a pathogenic hyper-responsive phenotype. The Local Lung Layer (right panel) outlines homing and tissue pathology. Stress-induced LYN downregulation converts SHIP-1 into a non-canonical scaffold complex that activates PI3K/Akt-STAT5-Trib1 signaling to lock macrophages in the pathogenic M2 phenotype, Subsequently driving aberrant subepithelial fibrosis, goblet cell hyperplasia, and airway smooth muscle (ASM) hypertrophy via pro-fibrotic mediator secretion. Clinical translation opportunities focusing on early warning via single-cell profiling and epigenetic memory resetting, alongside key research barriers regarding causality evidence gaps and pediatric toxicity concerns, are summarized in the bottom panel.

The upper horizontal gradient depicts a developmental timeline spanning prenatal life, infancy, early childhood, and school age. Prenatal bone-marrow (BM) trained immunity imprint (green curve) and congenital susceptibility established by maternal-placental signals (left panel) form a basal predisposing state that is insufficient to independently induce asthma pathology. Postnatal multi-hit exposures are required to trigger pathogenic cascades, driving progressive pulmonary remodeling and persistent inflammation (blue curve). The central Recursive Systemic-Local Loop panel illustrates the core pathogenesis: postnatal mucosal insults induce epithelial alarmin (IL-33, TSLP) release, which remotely reprograms BM hematopoietic stem/progenitor cells (HSPCs) through circulating mediators and promotes skewed monopoiesis. The resulting CD14^+^CD16^+^ trained monocytes traffic to the pulmonary niche and establish a positive feedback circuit that sustains long-term BM trained immune memory. The middle-right panel contrasts homeostatic resident alveolar macrophages (AMs) with pathogenic trained M2 bone-marrow-derived macrophages (BMDMs), which mediate subepithelial fibrosis and airway smooth muscle hypertrophy via TGF-β and PDGF-BB signaling. Model limitations (specific to severe T2-high allergic asthma) and translational perspectives regarding targeted innate immune memory resetting are summarized in the bottom-right panel.

## 2 Early-life exposure and myeloid plasticity: the lung-bone marrow axis

### 2.1 The “window of opportunity” and epithelial alarmins

The first 1,000 days of life represent a highly sensitive “critical window of opportunity” during which the developing immune system exhibits maximum plasticity and environmental susceptibility (14, 15). During this period, frequent environmental insults to the immature airway mucosal barrier—most notably respiratory syncytial virus or human rhinovirus infections, airborne allergens (e.g., HDM), and microbial dysbiosis—trigger the rapid secretion of epithelial alarmins—namely IL-33, TSLP, and IL-25—by damaged bronchial epithelial cells (4, 8, 16, 17). While these alarmins activate local mucosal cells, accumulating evidence suggests they also breach the compromised epithelial barrier to enter the systemic circulation, serving as endocrine-like messengers that connect distant organ systems (10). However, the precise kinetics and threshold concentrations required for systemic spillover remain incompletely defined in human infants, representing an important area for future investigation.

### 2.2 The lung-bone marrow axis: translating local insults to systemic imprints

Furthermore, immune programming traces its roots back to the prenatal period, where maternal-placental transmission of systemic immune signals and microbial metabolites shapes the foundational baseline of the fetal myeloid and hematopoietic compartments (18). This early prenatal environment can prematurely reprogram hematopoietic progenitors, creating a hyper-responsive congenital template (9). While the SPHPA framework specifically focuses on postnatal multi-hit threshold triggers during the first 1,000 days of life, this prenatal transgenerational priming provides the indispensable bedrock upon which postnatal mucosal insults act. While traditional models focus exclusively on local airway tissues, emerging translational evidence highlights the central role of remote hematopoietic organs in asthma pathogenesis (10). Pulmonary-derived alarmins, particularly IL-33, travel through the bloodstream to target hematopoietic stem and progenitor cells (HSPCs) within the bone marrow niche (10, 19). This sustained remote stimulation drives profound “myeloid skewing” of HSPCs, accelerating monopoiesis and imprinting progenitor cells with a pro-inflammatory developmental trajectory (20, 21). Although local inflammatory loops operate concurrently, this remote programming helps explain why transient early-life mucosal insults can translate into persistent, systemic immunological susceptibility, pre-activating myeloid cells before they ever home to the lung parenchyma (22). While directly isolating bone marrow HSCs from healthy human infants remains ethically constrained, human translational evidence from birth cohort studies—such as (9), which links maternal-neonatal immune variation to “trained innate immunity at birth”—provides crucial clinical backing, complemented by progenitor-level mechanistic insights from early-life viral infections (10) (**Figure 2**).

**Figure 2:**
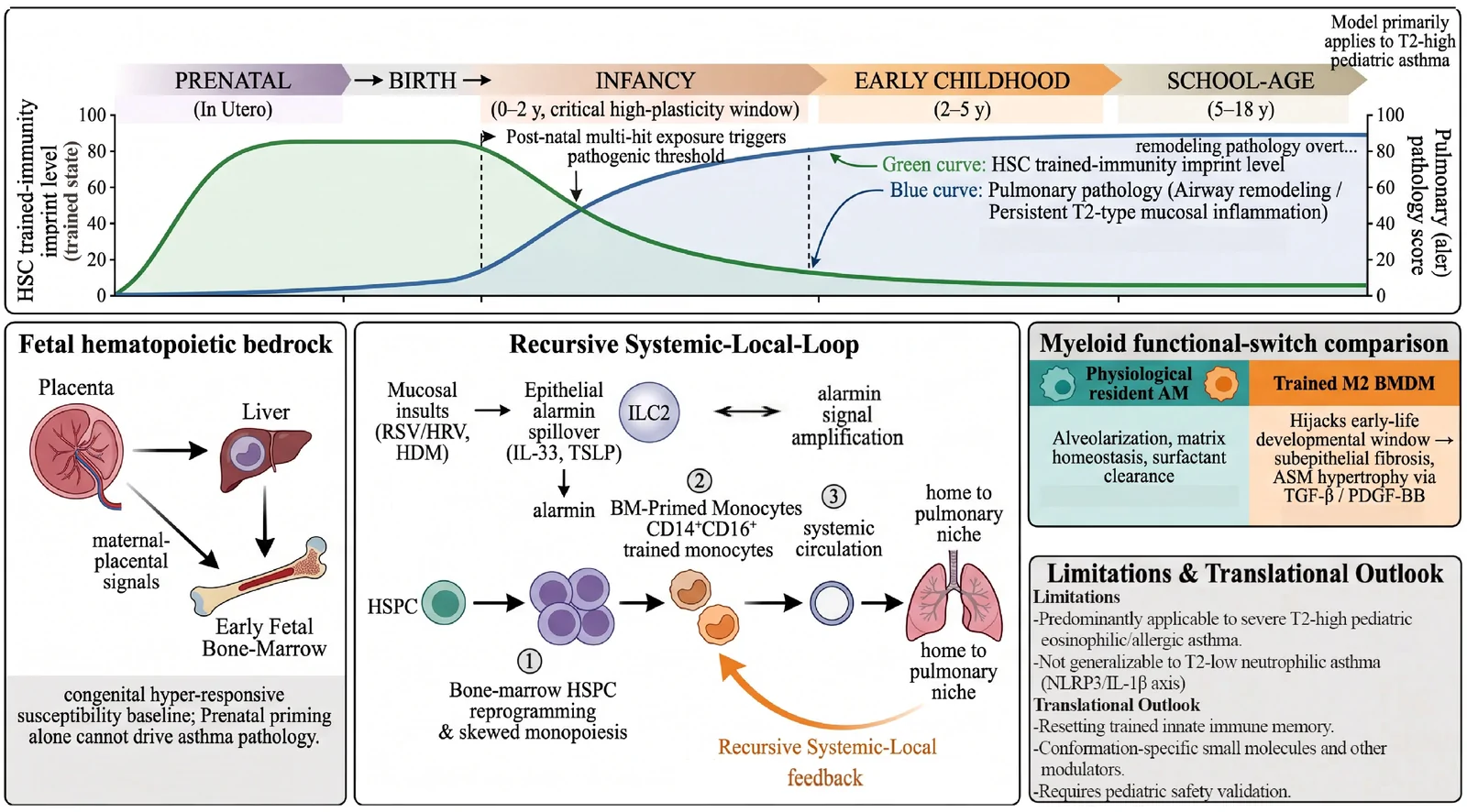
Developmental trajectory and recursive systemic-local immune crosstalk within the SPHPA framework spanning from prenatal life through childhood.

### 2.3 Trained immunity: metabolic-epigenetic couplers of myeloid memory

This bone marrow-level programming is driven by “trained immunity” (innate immune memory) (23, 24). During this process, HSPCs and newly formed monocytes undergo comprehensive metabolic rewiring (25). Studies demonstrate that early-life respiratory infections, combined with allergen sensitization, reprogram myeloid progenitors to rely on enhanced aerobic glycolysis and upregulated proline biosynthesis (26). This metabolic shift yields bioactive intermediates that serve as essential cofactors for nuclear chromatin-modifying enzymes (25). This metabolic-epigenetic coupling leads to the enrichment of activating histone modifications, such as H3K4me3 and H3K18la, on the promoters of pro-inflammatory genes (27, 28). Although H3K18la has been demonstrated primarily in vaccine-induced trained immunity models (27), its presence in circulating monocytes from asthmatic children requires direct clinical validation. These modifications maintain chromatin in a highly accessible configuration, ensuring that when trained monocytes eventually home to the lung, they mount a hyper-responsive, pathologic burst of inflammatory mediators (24, 29).

While this review centers on monocyte-macrophage-mediated trained immunity in severe pediatric asthma, it is important to acknowledge that other innate effectors, such as neutrophils and eosinophils, also undergo stimulus-driven functional reprogramming and exhibit innate immune memory features in asthmatic airways (30, 31). Nevertheless, given the unique systemic migratory capacity of monocytes and their central role in structural remodeling via the LYN-SHIP-1-Trib1 circuit, the subsequent sections will exclusively focus on monocyte-specific regulatory mechanisms.

## 3 Systemic monocyte pre-activation: the core pathological hub

Under the SPHPA framework, circulating monocytes are not passive, short-lived inflammatory effector cells. Instead, they act as the primary systemic vectors that harbor early-life bone marrow imprints and transport them directly to the lung microenvironment (13). This “functional priming” occurs across cellular, transcriptional, and post-translational dimensions.

### 3.1 Heterogeneity shift of circulating monocytes

Single-cell technologies have revealed significant phenotypic shifts in the peripheral blood monocytes of asthmatic children (32, 33). Pediatric clinical cohorts demonstrate a significant increase in the frequency of circulating CD14^+^CD16^+^ intermediate monocytes (13). Critically, the abundance of this intermediate population correlates negatively with FEV_1_ and positively with overall clinical asthma severity (13). This leftward shift in the peripheral monocyte pool directly reflects the bone marrow’s myeloid bias, serving as a robust systemic biomarker of the patient’s pathological immune status (34). However, given the high longitudinal variability of pediatric immune markers during acute exacerbations versus stable phases, the prognostic stability of these monocyte subsets warrants validation in larger, multi-center prospective longitudinal cohorts.

### 3.2 Transcriptional priming and the hyper-responsive state

Single-cell RNA sequencing (scRNA-seq) has provided initial evidence suggesting that circulating monocytes in asthmatic patients are transcriptionally primed prior to tissue recruitment. One recent study reported baseline upregulation of Type I interferon response genes (IRGs, such as *IFITM1, MX1, and ISG15*) and NF-κB target genes in peripheral blood monocytes from asthmatic children, though this finding is derived from a relatively small cohort (32). Additionally, transcriptomic analysis of bronchoalveolar lavage fluid from children experiencing acute asthma exacerbations revealed increased monocyte counts and the activation of infection-related signaling pathways (e.g., EIF2 and CXCR4 signaling) (35). While these findings support pre-activation, larger cohort studies are urgently needed to validate these transcriptional signatures and to determine whether comparable priming persists in peripheral blood monocytes during periods of disease stability, particularly given potential confounding effects of concurrent subclinical viral exposures (32, 35).

### 3.3 Post-translational control: SHIP-1 proteostasis and brake failure

The functional stability of circulating monocytes is heavily regulated at the post-translational level by key negative regulatory proteins (21). The lipid phosphatase SHIP-1 serves as a critical homeostatic brake (20). Studies have demonstrated that the conditional deletion of SHIP-1 solely in mature, tissue-resident myeloid cells is insufficient to induce chronic pulmonary inflammation (21). This finding underscores the fundamental premise that asthma pathogenesis requires systemic programming initiated at the bone marrow progenitor level rather than mere local adaptation (21). Concurrently with systemic imprinting, SHIP-1 protein stability is compromised via aberrant post-translational modifications (36). Clinical analyses indicate that a subset of patients with severe inflammatory diseases exhibit accelerated degradation of SHIP-1 via the ubiquitin-proteasome system (36). This rapid degradation deprives the monocyte of its endogenous brake on the PI3K pathway (21, 36). The resulting unchecked PI3K/Akt/mTOR activation works synergistically with transcriptional priming, locking the circulating monocyte into a highly pathogenic, pre-activated state (12, 36).

## 4 Trained innate immunity: epigenetic landscapes and metabolic reprogramming

The durability of trained innate immunity is maintained by a reciprocal feedback loop between metabolic flux and chromatin accessibility (24, 25). In childhood asthma, systemic immune programming co-opts specific metabolic pathways to establish permanent pro-inflammatory chromatin states in monocytes (22).

### 4.1 The metabolic-epigenetic feedback loop

Stimulation of monocytes by early-life pathogens or allergens triggers a metabolic shift from oxidative phosphorylation toward aerobic glycolysis (23, 24). This metabolic transition leads to the intracellular accumulation of specific bioactive metabolites that serve as essential substrates and cofactors for chromatin-modifying enzymes (25). The upregulation of proline biosynthesis in trained macrophages provides key energetic and biosynthetic support to sustain their primed state (26). Concurrently, increased glycolytic flux drives the accumulation of intracellular lactate (27). This lactate acts as a direct donor for H3K18la (27). H3K18la enrichment at the active enhancer and promoter regions of pro-inflammatory genes (e.g., *IL6, TNF*) locks these loci into a permanently accessible configuration (27, 28). Although H3K18la and related metabolic-epigenetic coupling events have been largely elucidated through vaccine-induced trained immunity models or murine systems (27), their precise contribution to circulating monocyte memory in pediatric asthma requires rigorous clinical verification. This metabolic-epigenetic coupling mechanistically explains how transient early-life environmental exposures translate into long-term innate immune hyper-responsiveness (28).

### 4.2 Metabolomic proof and the collapse of immune tolerance

This systemic metabolic reprogramming is clearly reflected in the plasma metabolome of pediatric asthma patients (37). Untargeted metabolomics shows that children with moderate-to-severe asthma possess significantly elevated circulating levels of lipids (ceramides, triglycerides) and carboxylic acids (lactate, aconitate), alongside depleted levels of amino acids (arginine, sarcosine) (37). While these systemic metabolomic signatures correlate robustly with clinical severity, determining whether they directly drive or merely reflect immune dysregulation warrants caution. Nevertheless, this signature exerts direct pathological effects on immune tolerance:

- Treg Dysfunction: Elevated circulating lactate and associated metabolites correlate with upregulated Notch4 expression and increased GDF15 secretion on circulating Treg cells (37). This Notch4 upregulation directly impairs the suppressive capacity of Tregs, destabilizing immunological tolerance (37).
- H3K4me3 Enrichment: Altered metabolic flux promotes the recruitment of histone methyltransferases to the promoters of IL6 and TNF, enriching the activating H3K4me3 mark and establishing the hallmark chromatin signature of trained immunity (28, 38).

Through this metabolic-epigenetic loop, monocytes are genetically and metabolically hardwired in the bone marrow to rapidly initiate and sustain a pro-inflammatory cascade upon homing to the lungs (29, 33).

## 5 Breakdown of the LYN-SHIP-1 signaling engine: the non-canonical scaffold flip

The loss of monocyte homeostatic control is driven by the breakdown and functional reconfiguration of core intracellular negative regulatory pathways (11). While multiple intersecting molecular circuits—such as A20-mediated master regulation of alarmin signaling (39), SREBP2-driven metabolic adaptation (40), and diverse macrophage subset interactions—co-shape the pediatric asthma immunophenotype, we propose the LYN-SHIP-1-Trib1 circuit as a central mechanistic paradigm that bridges systemic bone marrow imprinting with local structural remodeling.

### 5.1 LYN kinase: the gatekeeper of SHIP-1 balance

The non-receptor tyrosine kinase LYN serves as one of the earliest vital gatekeeper of inflammatory responses within this monocyte axis (41). Under physiological conditions, LYN maintains homeostatic balance by suppressing STAT3 activation, thereby limiting excessive SHIP-1 transcription and preventing its premature membrane translocation (2, 11).

However, under conditions of LYN impairment or deficiency, this regulatory gatekeeping mechanism collapses (2, 11), licensing uncontrolled STAT3 activation and prompting aberrant SHIP-1 localization (11). This abnormal spatial localization and abundance of SHIP-1 under LYN-deficient conditions initiates the downstream collapse of the signaling engine (41).

### 5.2 The “functional flip” of SHIP-1

Traditionally, SHIP-1 is characterized as a negative regulator of the PI3K pathway, hydrolyzing PI(3,4,5)P3 to PI(3,4)P2 to terminate Akt activation (20, 42). However, under stressful pulmonary microenvironments (e.g., severe viral infections or high oxidative stress), overexpressed SHIP-1 can undergo a non-canonical “functional flip” (11).

Instead of functioning as an active lipid phosphatase, SHIP-1 converts into a physical scaffold protein (11). It binds directly to the IL-6 receptor (IL-6R) and the cytoskeletal linker Ezrin, assembling into a multi-protein signaling complex on the inner leaflet of the cell membrane (11). This scaffold complex directly propagates PI3K/Akt and downstream NF-κB signaling, unleashing a hyper-inflammatory burst of TNF-α and IL-6 (11, 36). This functional inversion—from an inhibitory signaling brake to a pro-inflammatory scaffold—explains the clinical paradox of why some pediatric patients exhibit severe, uncontrolled pulmonary inflammation despite maintaining elevated levels of SHIP-1 protein (11, 21).

### 5.3 Proteolytic degradation and unchecked Akt flux

In alternative pathological contexts, the physical depletion of SHIP-1 protein via accelerated proteasomal degradation also contributes to signaling breakdown (36). When LYN remains functional but SHIP-1 is rapidly degraded, the monocyte loses its primary endogenous brake on PI3K activity (21, 36). While this proteolytic degradation pathway may operate independently of the aforementioned scaffold flip depending on the specific microenvironmental stressor, both mechanisms culminate in sustained, unchecked PI3K/Akt/mTOR signaling (20, 43). Working in tandem with histone lactylation-mediated chromatin opening, this unbridled signaling pathway drives the terminal differentiation of trained monocytes into highly pathogenic, alternatively activated macrophages (AAMs) (12, 26).

## 6 Downstream pathogenic cascade: the LYN-SHIP-1-Trib1 circuit and airway remodeling

Upon homing to the lung parenchyma, the pathogenic potential of primed circulating monocytes is fully unleashed during their differentiation and polarization into mature macrophages (44, 45). This section elucidates how the LYN-SHIP-1-Trib1 pathway coordinates M2-like macrophage polarization, thereby driving early airway remodeling independently of classical adaptive T2 inflammation.

### 6.1 The SHIP-1-STAT5-Trib1 circuit in myeloid fate-commitment

The polarization of recruited monocytes into pathogenic M2-like macrophages is governed by strictly defined intracellular signaling circuits (12, 34). Qin et al. (12) demonstrated that SHIP-1 regulates myeloid polarization via a distinct PI3K/Akt-STAT5-Trib1 signaling axis. Within this circuit, aberrant SHIP-1 activation upregulates the pseudokinase Trib1 (12). Trib1 acts as a central transcriptomic regulator, skewing the polarization of monocytes toward the alternatively activated (M2-like) macrophage lineage, characterized by the robust upregulation of markers such as CD206 and Arg1 (12, 34). When the LYN-SHIP-1 axis is disrupted or undergoes its non-canonical functional flip, this pathway becomes chronically activated (11, 12). This chronic activation forces recruited monocytes to differentiate into pathogenic macrophages, establishing a dominant pro-fibrotic signaling niche within the lung (34, 44).

### 6.2 Cellular crosstalk: macrophage-derived mediators and airway remodeling

These polarized, pathogenic macrophages release potent growth factors and cytokines that initiate deleterious crosstalk with resident airway structural cells and local immune compartments (46):

- Crosstalk with Epithelial Alarmins and ILC2s: Expanding beyond a purely myeloid-centric view, recent advances highlight that locally activated group 2 innate lymphoid cells (ILC2s) and adaptive Th2 networks operate in tight coordination with recruited macrophages. Environmental triggers breach epithelial integrity to release core alarmins (TSLP, IL-33, and IL-25) (47), which activate tissue-resident ILC2s as rapid early pioneers. Crucially, recent mechanistic evidence demonstrates a bidirectional feedback loop wherein M2 macrophage-derived extracellular vesicles are internalized by ILC2s, triggering their robust production of type 2 cytokines (IL-5 and IL-13) (48). Thus, systemic bone marrow imprinting and local epithelial-ILC2 loops form a self-reinforcing amplification network.
- ASM Phenotypic Switching: AAMs secrete elevated levels of Transforming TGF-β and PDGF-BB (3, 46). These mediators act directly on receptor tyrosine kinases expressed on ASM cells (41), stimulating a phenotypic switch from a resting contractile state to a synthetic and proliferative state (3). This phenotypic transition drives ASM hyperplasia, hypertrophy, and an acquired resistance to apoptosis (49).
- Subepithelial Fibrosis and RBM Thickening: Macrophage-derived TGF-β activates subepithelial fibroblasts, driving their transdifferentiation into active myofibroblasts. This accelerates extracellular matrix deposition, directly resulting in the thickening of the RBM (46).
- Goblet Cell Hyperplasia: Soluble factors secreted by AAMs and ILC2-derived cytokines act on airway epithelial progenitors, disrupting epithelial barrier integrity and promoting goblet cell metaplasia, which culminates in clinical mucus hypersecretion (28, 46).

### 6.3 The “remodeling-first” paradigm shift

This programmed, myeloid-driven remodeling cascade provides a mechanistic explanation for early-life asthma pathogenesis (5). The traditional “airway-centric” model, which views remodeling strictly as a late-stage consequence of chronic inflammation, fails to account for why children as young as 2.5 years of age display advanced RBM thickening and ASM hypertrophy (5).

During steady-state infancy, normal tissue-resident macrophages and physiological myeloid cells act as essential developmental ‘builders’—orchestrating alveolarization, clearing surfactant and apoptotic debris, and maintaining extracellular matrix homeostasis to support proper lung growth (43, 50). However, under the SPHPA framework, metabolic-epigenetic reprogramming induced by early-life multi-hit priming fundamentally subverts these physiological homeostatic functions. Rather than sustaining normal structural maturation, pathologically trained monocytes recruited from the bone marrow acquire a hyper-responsive, pro-fibrotic state, diverting alveolar development toward structural distortion, aberrant smooth muscle hypertrophy, and subepithelial matrix deposition via pathogenic TGF-β and PDGF-BB signaling (46).

Under our proposed SPHPA framework, this early structural divergence is not merely a localized downstream consequence of chronic inflammation, but represents a primary developmental pathology orchestrated by trained, pre-activated monocytes and their macrophage derivatives during a critical early-life window (5, 22). However, we acknowledge that while myeloid-driven remodeling plays a major initiating role, the relative contributions of early structural changes versus persistent inflammatory cascades may vary across different pediatric phenotypes. Nonetheless, this “remodeling-first” paradigm underscores the necessity of targeting the systemic monocyte-macrophage axis early in the disease course to preserve pediatric lung function trajectories (13, 34).

## 7 Clinical translation: therapeutic windows, epigenetic erasure, and biomarkers

Conceptualizing childhood asthma treatment as the systemic resetting of pathogenic innate immune memory is strongly supported by recent advances in trained immunity-based vaccines (51) and allergen-specific immunotherapy (23). Breakthroughs in single-cell transcriptomics and structure-based small-molecule design now make targeting the monocyte-macrophage axis a highly viable therapeutic strategy (22, 24).

### 7.1 Systemic effects of targeted biologics

Single-cell transcriptomics has profoundly expanded our understanding of the mechanistic reach of monoclonal antibody biologics (52). Recent studies demonstrate that the clinical efficacy of biologics like omalizumab (anti-IgE) (53) and dupilumab (anti-IL-4Rα) (54, 55) extends well beyond local mucosal tissues. Following biologic therapy, pediatric patients display a systemic phenotypic shift within their circulating monocyte pool (52). Specifically, there is a marked reduction in the frequency of classical monocytes expressing pro-inflammatory IL-1β and active NF-κB signatures, accompanied by the restoration of homeostatic S100A-expressing monocyte subsets (52). This systemic resetting of the circulating monocyte pool is likely a core mechanism through which these biologics improve long-term lung function trajectories (52, 53).

### 7.2 Steroid resistance: the monocyte MIF-ANXA1 axis

For the approximately 10% of pediatric patients who remain stubbornly resistant to standard ICS therapies (3, 56), identifying alternative molecular checkpoints within the monocyte lineage is critical (57). ScRNA-seq profiling reveals that steroid-resistant patients harbor a distinct transcriptional signature in their circulating CD14^+^ monocytes, characterized by the marked upregulation of chemokine genes (*CCR1, CCL2, CXCL2*) (57). At the molecular level, these cells exhibit downregulation of Annexin A1 (ANXA1) and its receptor FPR2, coupled with the upregulation of Macrophage Migration Inhibitory Factor (MIF) (57). This critical imbalance in the MIF-ANXA1 pathway actively prevents the glucocorticoid receptor from executing its anti-inflammatory transcriptional program, thereby driving steroid resistance (57, 58). For these patients, simply escalating ICS doses proves futile; instead, novel targeted interventions capable of restoring the MIF-ANXA1 pathway balance within the monocyte pool are urgently required (57).

### 7.3 SHIP-1 conformation-specific ligands as epigenetic erasers

Given that the LYN-SHIP-1 pathway can undergo a non-canonical functional flip to act as a pro-inflammatory scaffold under stressful microenvironments (11), traditional non-specific SHIP-1 agonists (such as AQX-1125) may carry unforeseen off-target risks in pediatric populations (11, 21, 42, 59), despite showing acceptable safety and efficacy profiles in adult mild-to-moderate asthma (42). Therefore, future clinical translation must be approached with extreme caution, as translating epigenetic and metabolic modulators into pediatric cohorts poses significant bottlenecks concerning safety profiles, spatiotemporal delivery precision, and model system limitations (60). Future drug development should prioritize conformation-specific small-molecule ligands engineered to prevent the deleterious scaffold transition while preserving or restoring the enzyme’s core lipid phosphatase activity (24, 61).

Recently, Jesudason et al. (61) developed a novel class of targeted SHIP1 ligands in neurodegenerative disease models, successfully demonstrating the feasibility of conformation-specific SHIP-1 modulation. Integrating these findings with broader cellular identity remodeling strategies—such as the four-dimensional technology toolbox proposed for metabolic and epigenetic reprogramming (60)—provides a translational framework to reset pathological myeloid memory. Although direct *in vivo* evidence in pediatric asthma remains speculative and requires rigorous validation, this cross-disciplinary proof-of-concept offers a promising strategy to arrest downstream airway structural remodeling at its fundamental source (5, 22).

### 7.4 Endotype specificity, model comparisons, and limitations

It is important to acknowledge the boundaries of the SPHPA framework. Specifically, the monocyte reprogramming mechanism and metabolic-epigenetic coupling described herein have been primarily characterized in T2-high (allergic, eosinophilic) pediatric asthma (26). By contrast, T2-low (neutrophilic) pediatric asthma exhibits a distinct innate architecture driven by NLRP3 inflammasome activation, IL-1β overproduction, and newly recruited CD206- monocytes rather than systemic trained immunity (62–64). Furthermore, compared to the classical “Airway-Centric Model” which confines pathogenesis to local mucosal inflammation and views remodeling as a late-stage consequence (4), the SPHPA model establishes a systemic bone marrow perspective to explain early remodeling. However, our framework should be viewed as a complementary paradigm rather than a universal explanation for all pediatric asthma endotypes, with its clinical generalizability awaiting large-scale prospective verification.

## 8 Conclusion and perspectives

This review integrates clinical, cellular, and biochemical evidence to substantiate a paradigm-shifting concept: childhood asthma is a systemic immune-programmed disorder initiated during early-life windows of maximum developmental plasticity (9, 10). Driven by a functional lung-bone marrow axis, early mucosal insults induce trained immunity and persistent metabolic-epigenetic imprints within hematopoietic progenitors (10, 23). This systemic programming pre-activates circulating intermediate monocytes—a pathological state actively maintained by the non-canonical functional flip and accelerated proteasomal degradation of the LYN-SHIP-1 gatekeeping pathway (11, 36). Upon homing to the lungs, these pre-activated cells hijack the LYN-SHIP-1-Trib1 pathway to polarize into pathogenic M2-like macrophages (12, 34), directly orchestrating early-life structural remodeling independently of, and prior to, classical T2 adaptive inflammation (5, 22).

To successfully translate the SPHPA model into clinical practice, while acknowledging current experimental and translational limitations, future research must prioritize three key imperatives:

- Multi-omic early detection: Developing non-invasive, predictive biomarker panels for high-risk wheezing infants by mapping circulating intermediate monocyte scRNA-seq profiles, MIF-ANXA1 ratios, and H3K18la enrichment (13, 38, 57), while accounting for longitudinal marker variability.
- Structural and biophysical characterization of the SHIP-1 scaffold: Resolving the three-dimensional conformational dynamics and binding pocket configurations of SHIP-1 during its functional flip with IL-6R and Ezrin, thereby enabling precision structure-based drug design (11).
- Epigenetic memory erasure and safety profiling: Advancing the rigorous pediatric safety profiling, spatiotemporal delivery precision, and model system validation for conformation-specific SHIP-1 ligands and cell-identity remodeling therapies, aiming to fundamentally reset pathological innate immune memory during critical early-life therapeutic windows (22, 23, 60, 61).

## References

[1] Global Initiative for Asthma . Asthma management and prevention for adults, adolescents and children 6–11 years. In: A Summary Guide for Healthcare Providers Wisconsin, USA: Global Initiative for Asthma; (2026). Available online at: ginasthma.org (Accessed August 19, 2026).

[2] Beavitt SJ Harder KW Kemp JM Jones J Smith A Brown C . Lyn-deficient mice develop severe, persistent asthma: Lyn is a critical negative regulator of Th2 immunity. J Immunol. (2005) 175:1867–75. doi: 10.4049/jimmunol.175.3.1867 16034130

[3] Filippo D Castagnoli R Brambilla I Leone M Marseglia GL Licari A . Management of severe asthma in children: current insights and future directions. Expert Rev Clin Immunol. (2025) 21:587–601. doi: 10.1080/1744666x.2025.2493698 40222821

[4] Mjösberg J Emgård J . The immunopathology of asthma. Lakartidningen. (2025) 122:24004. 40371567

[5] Castro-Rodriguez JA Saglani S Rodriguez-Martinez CE Oyarzun MA Fleming L Bush A . The relationship between inflammation and remodeling in childhood asthma: a systematic review. Pediatr Pulmonol. (2018) 53:824–35. doi: 10.1002/ppul.23968 29469196

[6] Hsu YL Huang HC Su TY Chen IL Wang C Liu Y . Early life factors associated with preschool wheezing in preterm infants. Children. (2021) 8:732. doi: 10.3390/children8090732 34572164 PMC8468763

[7] Mohan A Lugogo NL Hanania NA Reddel HK Akuthota P O'Byrne PM . Questions in mild asthma: an official American Thoracic Society research statement. Am J Respir Crit Care Med. (2023) 207:e77–96. doi: 10.1164/rccm.202304-0642st 37260227 PMC10263130

[8] Xiao Z Shi Y Zhao D Wang Y Gu Y Li Q . Triggering mechanisms of acute thunderstorm asthma: epithelial barrier disruption and immune dysregulation. Respir Res. (2026) 27:35. doi: 10.1186/s12931-026-03532-6 41652394 PMC12977724

[9] DeVries A McCauley K Fadrosh D Fujimura KE Stern DA Lynch SV . Maternal prenatal immunity, neonatal trained immunity, and early airway microbiota shape childhood asthma development. Allergy. (2022) 77:3617–28. doi: 10.1111/all.15442 35841380 PMC9712226

[10] Malinczak CA Lukacs NW Fonseca W . Early-life respiratory syncytial virus infection, trained immunity and subsequent pulmonary diseases. Viruses. (2020) 12:505. doi: 10.3390/v12050505 32375305 PMC7290378

[11] Li R Fang L Pu Q Lin P Hoggarth A Huang H . Lyn prevents aberrant inflammatory responses to Pseudomonas infection in mammalian systems by repressing a SHIP-1-associated signaling cluster. Signal Transduct Target Ther. (2016) 1:16032. doi: 10.1038/sigtrans.2016.32 29263906 PMC5661651

[12] Qin S Li J Zhou C Privratsky B Schettler J Deng X . SHIP-1 regulates phagocytosis and M2 polarization through the PI3K/Akt-STAT5-Trib1 circuit in Pseudomonas aeruginosa infection. Front Immunol. (2020) 11:307. doi: 10.3389/fimmu.2020.00307 32256487 PMC7093384

[13] Mostafa R Al-Diwany O Hammad R Hamed DH Ali S Hassan M . The role of monocytes and natural killers' immunophenotypic subsets in bronchial asthma in children. J Asthma. (2023) 60:244–54. doi: 10.1080/02770903.2022.2043361 35171740

[14] Hoskinson C Petersen C Turvey SE . How the early life microbiome shapes immune programming in childhood asthma and allergies. Mucosal Immunol. (2025) 18:26–35. doi: 10.1016/j.mucimm.2024.12.005 39675725

[15] Diaconu ID Gheorman V Grigorie GA Gheonea C Tenea-Cojan TS Mahler B . A comprehensive look at the development of asthma in children. Children. (2024) 11:581. doi: 10.3390/children11050581 38790577 PMC11120211

[16] Lau S Illi S Sommerfeld C Niggemann B Bergmann R von Mutius E . Early exposure to house-dust mite and cat allergens and development of childhood asthma: a cohort study. Lancet. (2000) 356:1392–7. doi: 10.1016/s0140-6736(00)02842-7 11052581

[17] Jia XQ Lu L Lou SY Han SY Deng LL Wu WS . Association between early-life respiratory syncytial virus infection and the risk of asthma: a meta-analysis. J Thorac Dis. (2025) 17:9980–96. doi: 10.21037/jtd-2025-1536 41376982 PMC12688565

[18] Lynch SV Vercelli D . Microbiota, epigenetics, and trained immunity: convergent drivers and mediators of the asthma trajectory from pregnancy to childhood. Am J Respir Crit Care Med. (2021) 203:802–8. doi: 10.1164/rccm.202010-3779PP 33493428 PMC8017579

[19] Subbarao P Anand SS Becker AB Befus AD Brauer M Brook JR . The Canadian Healthy Infant Longitudinal Development (CHILD) Study: examining developmental origins of allergy and asthma. Thorax. (2015) 70:998–1000. doi: 10.1136/thoraxjnl-2015-207246 26069286

[20] So EY Sun C Reginato AM Dubielecka PM Ouchi T Liang OD . Loss of lipid phosphatase SHIP1 promotes macrophage differentiation through suppression of dendritic cell differentiation. Cancer Biol Ther. (2019) 20:201–11. doi: 10.1080/15384047.2018.1523846 30277839 PMC6343724

[21] Maxwell MJ Srivastava N Park MY Tsantikos E Engelman RW Kerr WG . SHIP-1 deficiency in the myeloid compartment is insufficient to induce myeloid expansion or chronic inflammation. Genes Immun. (2014) 15:233–40. doi: 10.1038/gene.2014.9 24598798

[22] Laubhahn K Schaub B . From preschool wheezing to asthma: immunological determinants. Pediatr Allergy Immunol. (2023) 34:e14038. doi: 10.1111/pai.14038 37877843

[23] Martín-Cruz L Sevilla-Ortega C Angelina A Domínguez-Andrés J Netea MG Subiza JL . From trained immunity in allergy to trained immunity-based allergen vaccines. Clin Exp Allergy. (2023) 53:145–55. doi: 10.1111/cea.14261 36494877

[24] Martín-Cruz L Palomares O . Allergen-specific immunotherapy and trained immunity. Allergy. (2025) 80:677–89. doi: 10.1111/all.16423 39641571 PMC11891420

[25] Patil ND Turner JD Desai MS Zimmer J Sharma R Gupta S . Trained through generations. Cell Mol Immunol. (2022) 19:653–4. doi: 10.1038/s41423-022-00846-5 35277673 PMC8913860

[26] Li H Ma L Li W Zheng B Wang J Chen S . Proline metabolism reprogramming of trained macrophages induced by early respiratory infection combined with allergen sensitization contributes to development of allergic asthma in childhood of mice. Front Immunol. (2022) 13:977235. doi: 10.3389/fimmu.2022.977235 36211408 PMC9533174

[27] Ziogas A Novakovic B Ventriglia L Galang N Tran KA Li W . Long-term histone lactylation connects metabolic and epigenetic rewiring in innate immune memory. Cell. (2025) 188:2992–3012.e16. doi: 10.1016/j.cell.2025.03.048 40318634

[28] Wang Y Xiang Z Zhu J . Epigenetic regulation of airway epithelial barrier dysfunction in pediatric asthma: mechanistic insights and therapeutic potential. Front Med (Lausanne). (2025) 12:1652746. doi: 10.3389/fmed.2025.1652746 41613305 PMC12848801

[29] Wang Y Ge F Wang J Li H Zheng B Li W . Mycobacterium bovis BCG given at birth followed by inactivated respiratory syncytial virus vaccine prevents vaccine-enhanced disease by promoting trained macrophages and resident memory T cells. J Virol. (2023) 97:e0176422. doi: 10.1128/jvi.01764-22 36779760 PMC10062174

[30] Teague WG Lawrence MG Shirley DT Garrod AS Early SV Payne JB . Lung lavage granulocyte patterns and clinical phenotypes in children with severe, therapy-resistant asthma. J Allergy Clin Immunol Pract. (2019) 7:1803–12. doi: 10.1016/j.jaip.2018.12.027 30654199 PMC6612461

[31] Moorlag SJC Rodriguez-Rosales YA Gillard J Fanucchi S Theunissen K Novakovic B . BCG vaccination induces long-term functional reprogramming of human neutrophils. Cell Rep. (2020) 33:108387. doi: 10.1016/j.celrep.2020.108387 33207187 PMC7672522

[32] Goss K Grant ML Guglani L Miller H Woods EJ Caldwell C . Single cell transcriptional profiling of monocytes from asthma patients show a predisposition for an inflammatory response. Cytotherapy. (2026) 28:101967. doi: 10.1016/j.jcyt.2025.07.008 41263748

[33] Read JF Serralha M Armitage JD Martinez R Zhang Y Chen L . Single cell transcriptomics reveals cell type specific features of developmentally regulated responses to lipopolysaccharide between birth and 5 years. Front Immunol. (2023) 14:1275937. doi: 10.3389/fimmu.2023.1275937 37920467 PMC10619903

[34] Kuo CH Tsai ML Li CH Hsiao HP Chao MC Lee MS . Altered pattern of macrophage polarization as a biomarker for severity of childhood asthma. J Inflammation Res. (2021) 14:6011–23. doi: 10.2147/jir.s319754 34819741 PMC8608023

[35] Li H Wang H Sokulsky L Liu S Yang R Liu X . Single-cell transcriptomic analysis reveals key immune cell phenotypes in the lungs of patients with asthma exacerbation. J Allergy Clin Immunol. (2021) 147:941–54. doi: 10.1016/j.jaci.2020.09.032 33039479

[36] Ngoh EN Kerr WG . Altered SHIP-1 protein stability in a subset of IBD patients. Inflammation Bowel Dis. (2014) 20:S104.

[37] Crestani E Harb H Benamar M Kaddurah-Daouk R Phipatanakul W Chatila TA . Metabolomic and immunological signatures of asthma severity in children. Pediatr Allergy Immunol. (2025) 36:e70242. doi: 10.1111/pai.70242 41195700 PMC12636052

[38] Li Y Jones K Ober C Starling AP Gower WA Bacharier LB . Newborn blood DNA methylation and childhood asthma: findings from the ECHO program. Int J Epidemiol. (2025) 54:dyaf067. doi: 10.1093/ije/dyaf067 40420521 PMC12106279

[39] Holgado A Liu Z Aidarova A Mueller C Haegman M Driege Y . A20 is a master switch of IL-33 signaling in macrophages and determines IL-33-induced lung immunity. J Allergy Clin Immunol. (2023) 152:244–56.e4. doi: 10.1016/j.jaci.2023.02.026 36898482

[40] Wu Y Zheng K Dong LL Gao S Wu Y Liu Z . SREBP2 promotes macrophage alternative activation and allergic airway inflammation independent of cholesterol biosynthesis. Cell Death Dis. (2026) 17:179. doi: 10.1038/s41419-026-08910-y 42236481 PMC13448065

[41] Wong WS . Inhibitors of the tyrosine kinase signaling cascade for asthma. Curr Opin Pharmacol. (2005) 5:264–71. doi: 10.1016/j.coph.2005.01.009 15907913

[42] Leaker BR Barnes PJ O'Connor BJ Ali FY Tam P Neville J . The effects of the novel SHIP1 activator AQX-1125 on allergen-induced responses in mild-to-moderate asthma. Clin Exp Allergy. (2014) 44:1146–53. doi: 10.1183/13993003/erj.42.suppl_57.p1592 25040039

[43] Ntontsi P Photiades A Zervas E Xanthou G Samitas K Karadag B . Genetics and epigenetics in asthma. Int J Mol Sci. (2021) 22:2412. doi: 10.3390/ijms22052412 33673725 PMC7957649

[44] Fricker M Gibson PG . Macrophage dysfunction in the pathogenesis and treatment of asthma. Eur Respir J. (2017) 50:1700196. doi: 10.1183/13993003.00196-2017 28899935

[45] Funes SC Rios M Escobar-Vera J Kalergis AM Soto-Quirós M Bustos P . Implications of macrophage polarization in autoimmunity. Immunology. (2018) 154:186–95. doi: 10.1111/imm.12910 29455468 PMC5980179

[46] Hsieh A Assadinia N Hackett TL . Airway remodeling heterogeneity in asthma and its relationship to disease outcomes. Front Physiol. (2023) 14:1113100. doi: 10.3389/fphys.2023.1113100 36744026 PMC9892557

[47] Zhu Z Li Q Hua B . The airway epithelial-immune axis: mechanisms and therapeutic implications. Front Immunol. (2026) 17:1891760. doi: 10.3389/fimmu.2026.1891760 42568577 PMC13447427

[48] Lv K Zhang Y Yin G Li X Zhong M Zhu X . Extracellular vesicles derived from lung M2 macrophages enhance group 2 innate lymphoid cells function in allergic airway inflammation. Exp Mol Med. (2025) 57:1202–15. doi: 10.1038/s12276-025-01465-6 40451928 PMC12229531

[49] Chetty A Nielsen HC . Targeting airway smooth muscle hypertrophy in asthma: an approach whose time has come. J Asthma Allergy. (2021) 14:539–56. doi: 10.2147/jaa.s280247 34079293 PMC8164696

[50] Pervizaj-Oruqaj L Ferrero MR Matt U Herold S . The guardians of pulmonary harmony: alveolar macrophages orchestrating the symphony of lung inflammation and tissue homeostasis. Eur Respir Rev. (2024) 33(172):230263. doi: 10.1183/16000617.0263-2023 38811033 PMC11134199

[51] Kim W Jeoung D . How to employ trained immunity and trained immunity-based vaccines to inhibit allergic inflammation. Vaccines. (2026) 14:268. doi: 10.3390/vaccines14030268 41893804 PMC13030827

[52] Park K Lee JH Shin E Jang HY Song WJ Kwon HS . Single-cell RNA sequencing reveals transcriptional changes in circulating immune cells from patients with severe asthma induced by biologics. Exp Mol Med. (2024) 56:2755–62. doi: 10.1038/s12276-024-01368-y 39672815 PMC11671581

[53] Phipatanakul W Mauger DT Guilbert TW Bacharier LB Durrani S Jackson DJ . Preventing asthma in high risk kids (PARK) with omalizumab: design, rationale, methods, lessons learned and adaptation. Contemp Clin Trials. (2021) 100:106228. doi: 10.1016/j.cct.2020.106228 33242697 PMC7887056

[54] Dijk YE Rutjes NW Golebski K Şahin H Hashimoto S Maitland-van der Zee AH . Developments in the management of severe asthma in children and adolescents: focus on dupilumab and tezepelumab. Paediatr Drugs. (2023) 25:677–93. doi: 10.1007/s40272-023-00589-4 37658954 PMC10600295

[55] Saxena S Rosas-Salazar C Fitzpatrick A Bacharier LB Brown M Chen R . Biologics and severe asthma in children. Curr Opin Allergy Clin Immunol. (2023) 23:111–8. doi: 10.1097/aci.0000000000000880 36730217

[56] Pijnenburg MW Frey U Jongste JCD Saglani S Martinez F Reddel H . Childhood asthma: pathogenesis and phenotypes. Eur Respir J. (2022) 59:2100731. doi: 10.1183/13993003.00731-2021 34711541

[57] Kwon H Kang M Jeong S Chae M Lee HS Lee BH . Single-cell transcriptomic profiling reveals a distinct monocyte MIF/ANXA1 signature associated with poor responsiveness to ICS. Allergy Asthma Immunol Res. (2025) 17:658–67. doi: 10.4168/aair.2025.17.5.658 41044838 PMC12511794

[58] Alonso AM Saglani S . Mechanisms mediating pediatric severe asthma and potential novel therapies. Front Pediatr. (2017) 5:154. doi: 10.3389/fped.2017.00154 28725641 PMC5497140

[59] Stenton GR Mackenzie LF Tam P Cross JL Harwig C Raymond J . Characterization of AQX-1125, a small-molecule SHIP1 activator: part 2. Efficacy studies in allergic and pulmonary inflammation models *in vivo*. Br J Pharmacol. (2013) 168:1519–29. 10.1111/bph.12038 PMC3596655 23121409

[60] Wang J Chen M Wei J Chen X Wang C Feng H . Reprogramming the wound microenvironment: identity remodeling strategies for fibroblasts, keratinocytes, and macrophages. Front Immunol. (2026) 17:1892931. doi: 10.3389/fimmu.2026.1892931 42548532 PMC13429392

[61] Jesudason CD Rangel-Barajas C Beach CJ Beck DE Caballero-Floran IH Clayton WB . Optimization and characterization of SHIP1 ligands for cellular target engagement and activity in Alzheimer's disease models. In: Biorxiv (2026). doi: 10.64898/2025.12.31.697127 PMC13337215 42059452

[62] Niessen NM Baines KJ Simpson JL Scott HA Qin L Gibson PG . Neutrophilic asthma features increased airway classical monocytes. Clin Exp Allergy. (2021) 51:305–17. doi: 10.1111/cea.13811 33301598

[63] Niessen NM Gibson PG Simpson JL Scott HA Baines KJ Fricker M . Airway monocyte modulation relates to tumour necrosis factor dysregulation in neutrophilic asthma. ERJ Open Res. (2021) 7:00131–2021. doi: 10.1183/23120541.00131-2021 34291112 PMC8287135

[64] Hinks TSC Levine SJ Brusselle GG . Treatment options in type-2 low asthma. Eur Respir J. (2021) 57:2000528. doi: 10.1183/13993003.00528-2020 32586877 PMC7116624

